# Probabilistic simulation of big climate data for robust quantification of changes in compound hazard events

**DOI:** 10.1016/j.wace.2022.100522

**Published:** 2022-12

**Authors:** Theodoros Economou, Freya Garry

**Affiliations:** a Climate and Atmospheric Research Centre, The Cyprus Institute, Nicosia, Cyprus; b Met Office, UK Climate Resilience, Exeter, United Kingdom

**Keywords:** Climate change, GAMs, Bayesian smoothing, Stochastic simulation, Space–time model

## Abstract

Understanding changes in extreme compound hazard events is important for climate mitigation and policy. By definition, such events are rare so robust quantification of their future changes is challenging. An approach is presented, for probabilistic modelling and simulation of climate model data, which is invariant to the event definition since it models the underlying weather variables. The approach is based on the idea of a ‘moving window’ in conjunction with Generalised Additive Models (GAMs) and Bayesian inference. As such, it is robust to the data size and completely parallelizable, while it fully quantifies uncertainty allowing also for comprehensive model checking. Lastly, Gaussian anamorphosis is used to capture dependency across weather variables. The approach results in probabilistic simulations to enable extrapolation beyond the original data range and thus robust quantification of future changes of rare events. We illustrate by application to daily temperature, humidity and precipitation from a regional climate model.

## 0 Introduction

The analysis and quantification of climate change impacts involves the use of data in the form of climate model output. For instance, efforts such as the Coupled Model Intercomparison Project Phase 6 or CMIP6 makes use of 134 climate models from 53 modelling centres (CMIP6, 2021). To better understand and interpret the potential impact of the future climate projections, interest often lies in the probability of compound events (hazards) such as heat waves rather than the individual “raw” variables e.g. temperature. Questions such as “what is the probability that event W= temperature exceeds 24 °C for three consecutive days over the UK will occur and how will this change in the future?” are an example of the definition of such events. In addition, events can be compounded by being multi-variate, i.e. more than one meteorological variable is involved (Zscheischler et al., 2020). One example is the occurrence of “warm–dry” days, defined as days for which temperature is above a baseline average and precipitation is below a baseline average.

Interest usually lies in events that are rare and therefore in some sense extreme. Despite the amount of available data, say in CMIP6, quantifying the occurrence of rare events may result in very high uncertainty — depending of course on the rarity of the event. The frequency of warm–dry days for instance might not cause an issue, however the frequency of W might be more problematic: computing the frequency of such events empirically from climate model simulations – which essentially amounts to counting them – assumes there is enough data to begin with. What if for instance the event W never occurs in a particular grid cell over a 20-year historical period from a particular model run? Do we believe that the probability of W occurring is actually zero? Of course the problem is exacerbated when more than one weather/climate variable is involved, noting that in reality hazard events are a very complex interaction of physical processes (Leonard et al., 2014). This empirical approach to quantification of event probability is reliable only when one can safely assume there is an adequate amount of data.

An appealing way of remedying this problem is by statistical (probabilistic) modelling of the climate model output. Assuming that such probabilistic models can reliably capture the statistical properties of the climate model data, they can then be used to stochastically simulate as many realisations as necessary of the original raw variables. Then, quantification of rare and/or compound events is performed by computing the associated frequency of such events from the many realisations (e.g. Dodd et al. (2021)). Such an approach is appealing because it is invariant to the definition of the compound event — since the raw variables are the ones being modelled.

For such an approach to be of practical use however, certain requirements have to be taken into account. Firstly, the method needs to be scalable, i.e. robust to the size of the data. This is extremely important with the continuously increasing resolution of climate models and the multivariate nature of compound events. Second but equally important, the approach needs to be interpretable otherwise the utility of its outputs cannot realistically be quantified. We classify interpretability in two ways: (a) the method must be transparent and therefore reproducible and comprehensible by its user and (b) uncertainty must be fully quantified and so the method needs to be probabilistic. If we are to simulate events, we must be more uncertain about ones further away from the range of the data. We must be more uncertain about future changes, when for instance we compare 10-year versus 20-year periods since in the latter case there are more data. Third, we must be able to fully assess the ability of the approach to capture the nature of the data in terms of statistical properties such as tail behaviour, spatial structure, temporal correlation and so on. We argue that this can only be done rigorously if the simulations are probabilistic. Fourth, the approach must be flexible enough to be applicable across a range of meteorological variables with potentially very different statistical properties/distributions.

In this paper, we present an approach motivated by these requirements. The method is based on using the well-established ideas of a moving window and of Generalised Additive Models utilising penalised smoothing splines. A Bayesian interpretation of the method provides full quantification of uncertainty and therefore posterior predictive model checking. We demonstrate that the method is both scalable and paralleliseable and we present it in quantifying changes in regional climate model output over the UK. Due to the simplicity of its components, it can be argued that the approach is highly interpretable and robust to the choice of variables, and we illustrate this using temperature, relative humidity and precipitation — variables which are known to have very different statistical behaviour. Furthermore, we use the concept of Gaussian anamorphosis to capture dependence across climate variables and show this by quantifying future changes in compound events such as warm–dry days in the UK.

## 1 Background

Statistical analysis and modelling of climate model output is a well-established concept and includes methods for downscaling (Mosaffa et al., 2020), bias correction (Volosciuk et al., 2017) and post-processing (Wilks, 2019) just to name a few. The idea that we can mathematically describe weather and climate data using a probability distribution is particularly appealing for impact studies. The probability distribution can be used to extrapolate to values outside the range of the original data. Assuming it fully quantifies the associated uncertainties, it can be used to formally assess the likelihood of events, the magnitude of (future) changes and to produce stochastic simulations of the modelled variable(s).

### 1.1 Stochastic simulation for hazard event quantification

Hazard events often have very different temporal and spatial scales compared to the variables they are functions of. For instance, from daily mean temperature $y_{t}$ we could define $z_{t}=\left(y_{t-1}\text{ \& }y_{t}\text{ \& }y_{t+1}\right)>24$ which is an event that occurs very rarely. A 10-year simulation of $y_{t}$ may well be reduced to a handful of occurrences of $z_{t}$. Being able to stochastically simulate plausible realisations of $y_{t}$ can provide thousands of 10-year periods to obtain a much larger sample size of $z_{t}$ occurrence. This approach is akin to stochastic weather generators, and has long been utilised for example, by the water industry to quantify flood/drought risk (Dawkins et al., 2022) as well as the reinsurance sector for estimating natural hazard risk (Youngman and Economou, 2017).

The procedure has the added benefit of being invariant to the definition of the hazard, since the simulation is of the original raw variable. We could for example change the definition $z_{t}$ to a different function of $y_{t}$ and not require a different statistical model, noting that the event definition itself is a complex process (Bevacqua et al., 2021).

This simulation approach is often termed Monte Carlo simulation, since in practice it is akin to the method of Monte Carlo numerical integration (Wilks, 2019). The idea is simple albeit elegant. All that one needs to do is find a probability distribution that retains the properties of the original data and then stochastically simulate from this distribution many times. The more rare the event is, the more simulations are needed. It is vital however to ensure that the simulated data sets are statistically similar to the original data.

Note that the idea of using stochastic weather generators for quantifying hazard events and the associated effects of climate change is well-established. For instance, Fatichi et al. (2011) use a physically-based stochastic rainfall generator in conjunction with change factors to project future changes. Moreover, Paschalis et al. (2013) use a physically motivated storm–rainfall generator to simulate rainfall at very high spatio-temporal resolution. More recently, Li and Babovic (2019) have proposed an approach to stochastically simulate weather variables across different spatial locations, where the cross-correlation between variables and space is modelled by copulas. The approach we propose here uses the concept of stochastic weather generators to simulate climate output rather than observational data, a relatively more straightforward task since space–time coverage is guaranteed and the usual flaws in observed data are not an issue. On the other hand the method we present is multivariate (using empirical copulas) and robust to the choice of variables, as well as to the size of the data.

### 1.2 Statistical modelling challenges

Statistical modelling can be used to learn the appropriate probability distribution that describes the nature of the data. The primary difficulty lies in finding a statistical model that compromises complexity and scalability. Complexity is necessary as it provides the appropriate flexibility to adequately capture the nature of the data, albeit at computational cost. Moreover, even simple methods such as linear regression have limits to how well they scale with really big data sets, typical of those being outputted by climate models today.

There are a few things to consider when defining a statistical model for weather data. Firstly, for a single variable (e.g., temperature) we require that the spatial, temporal and also space–time structure are adequately captured. This requirement alone is enough to warrant rather expensive and complex statistical methods, such as Gaussian Process models or Markov Random Field based approaches (Cressie and Wikle, 2011, Banerjee et al., 2014). The situation is exacerbated for more than one variable because of cross-correlation, say between temperature and precipitation. Second, we must ensure that the marginal properties of the modelled variable are well captured. Temperature for instance may be adequately described by a Gaussian distribution, but rainfall is known to have very heavy tails and a probability mass at zero, whereas relative humidity is a percentage and thus bounded from above and below.

A further challenge is to have a unifying method with which to assess that the above properties of the data have been adequately captured, preferably without adding to the computational burden. Model checking is an important part of any data modelling approach, but here extra emphasis needs to be placed on this aspect. We must have confidence that the stochastically simulated data closely resemble the original data, otherwise the estimated impact is biased. E.g, if spatial structure is not well-captured because spatial correlation has been underestimated, then the extremes of any spatially aggregated hazard event will be underestimated. In the following section we describe an approach to deal with these challenges.

## 2 Statistical framework

### 2.1 Moving window

The biggest challenge with big data is scalability. One simple but arguably effective way to deal with big data is to subset them. Given the space–time structures in climate model output, we propose here to use a moving window approach over subspaces of the space–time domain. A moving window concept is by definition very scalable — it lies at the heart of modern machine learning methods such as random forests (Murphy, 2012). In its most basic form, a moving window predicts a response variable $y_{j}$ from data $y_{i}$ (i=1,…,n) as a function of some covariate $x_{j}$ (e.g., time) as

$${\overset{\sim }{y}}_{j}=\overset{n}{\underset{i=1}{\sum }}w\left(\left|x_{i}-x_{j}\right|\right)y_{i}$$

where the weight function w() typically gives less contribution to points $x_{i}$ that are further from the “window” centre $x_{j}$ — usually zero beyond certain distance. Various flavours of this approach have been suggested, with a plethora of choices for w(). The approach can be extended to include a model being fitted in each window (e.g., regression) to further increase flexibility and allow more straightforward uncertainty estimation for the predicted $y_{j}$ (e.g. Murphy (2012) chapter 14).

**Fig. 1:**
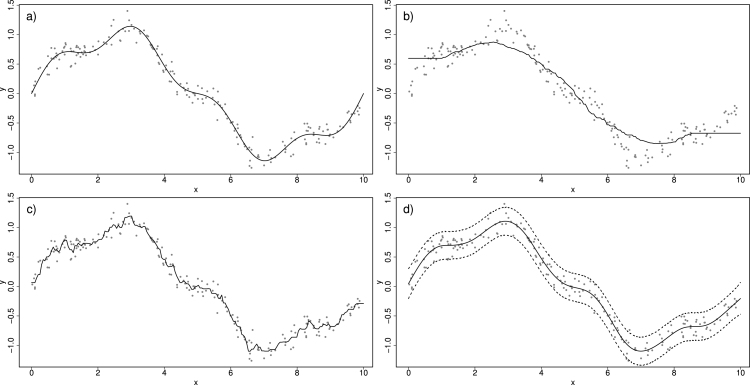
Simulated data (a) along with a moving average estimate of the trend where window size is based on m=5 neighbours in (b) and m=50 neighbours in (c). A Normal GAM estimate with 95% prediction intervals is shown in (d).

The size of the window is important: too small leads to over-fitting (the extreme being a single data point per window) and too large might not be flexible enough to capture structure in the data. Fig. 1a shows 200 simulated data points assuming the smooth trend shown is the “true relationship”, along with some random Normal deviation with constant variance about this truth. Fig. 1 also shows an example of a moving window where $w\left(\left|x_{i}-x_{j}\right|\right)=1/m$ if $\left|x_{i}-x_{j}\right|$ is within the smallest m values over all i. So prediction $\overset{\sim }{y}$ is the average of the m nearest (in x space) $y_{i}$ values. Fig. 1b shows the m=5 case where the prediction is clearly too wiggly. Fig. 1c refers to m=50 where the trend is clearly missed. Data-driven estimation of the window size is possible (e.g., Schucany, 1995, Ruppert, 1997, Prewitt and Lohr, 2006) albeit at a computational cost that takes away the appeal of moving windows as a “cheap” approach.

Here, we consider using moving windows in a way that is robust to window size. The idea is to have a data model inside each window that can flexibly capture structure in the data (in each window) at low computational cost. Flexibility is crucial, for if the window size is too large, then data points deemed too far (in covariate space) from the prediction point will have less influence in the prediction. We propose the use of Generalised Additive Models (GAMs) that utilise penalised splines as an option.

### 2.2 Generalised additive models

GAMs are semi-parametric regression models (Wood, 2017) where the mean of response variable $y_{i}$ is defined as a smooth function of one or more covariates $x_{i}$. For a single $x_{i}$, the general form of a GAM is

(1) $$y_{i}∼p\left(y_{i};\mu \left(x_{i}\right),\phi \right)$$

(2) $$g\left(\mu \left(x_{i}\right)\right)=f\left(x_{i}\right)$$

where $\mu \left(x_{i}\right)$ is the mean of the probability distribution p(⋅), and ϕ are parameters of the distribution that do not depend on $x_{i}$. The function f(⋅) is defined by regression splines (e.g. cubic splines) so it is a linear combination:

(3) $$f\left(x_{i}\right)=\overset{J}{\underset{j=1}{\sum }}\beta_{j}b_{j}\left(x_{i}\right)$$

where $\beta_{j}$ are unknown coefficients and $b_{j}\left(\cdot \right)$ are simple basis functions. Such GAMs as implemented in the R package mgcv (Wood, 2011) also include a penalty for when J (conventionally called the “number of knots”) is chosen to be too large, which would result in f(⋅) being too “wiggly” and thus the model over-fitting the data. The aim is to choose J to be larger than necessary and then allow the penalisation to prohibit over-fitting.

As an example, we implement the following GAM in mgcv to the data in Fig. 1a:

(4) $$y_{i}∼N\left(\mu_{i},\sigma^{2}\right)$$

(5) $$\mu \left(x_{i}\right)=f\left(x_{i}\right)$$

where f(x) is defined by cubic splines with J=29 knots. The penalised log-likelihood for $\beta ={\left(\beta_{1},\ldots ,\beta_{K}\right)}^{\prime }$ and $\sigma^{2}$, with data $y={\left(y_{1},\ldots ,y_{n}\right)}^{\prime }$ in this example can be written concisely as:

(6) $$\ell \left(\beta ,\sigma^{2};y\right)\propto -n\log\left(\sigma \right)-\overset{n}{\underset{i=1}{\sum }}\frac{1}{2\sigma^{2}}{\left(y_{i}-f\left(x_{i}\right)\right)}^{2}-\lambda \beta^{\prime }S\beta$$

where λ is the penalty parameter and S is a penalty matrix that relates to a quadratic penalty (on β). S is computed prior to model fitting and depends on the choice of basis functions. For cubic splines the elements of S relate to an approximation of the second derivative of $f\left(x_{i}\right)$ as well as any constraints (e.g., f(x) being centred on zero). After penalisation, only about 15 out of the 29 knots were needed to define the estimated function $f\left(x_{i}\right)$ shown depicted in Fig. 1d.

Penalised maximum likelihood is used to estimate coefficients $\beta_{j}$ and any hyperparameters ϕ (e.g., $\sigma^{2}$ in (4)). The amount of penalisation is governed by λ which is estimated from the data as a compromise between out-of-sample and in-sample predictive skill (Wood, 2017). In practice, implementing GAMs in mgcv (at least for small data sets) is extremely efficient.

However, recall that the goal here is to be able to simulate values of $y_{i}$ in such a way that all associated uncertainty is quantified. The issue then is that when it comes to predicting $y_{i}$ values we do not readily obtain a predictive distribution that captures both the uncertainty in estimating the parameters (epistemic uncertainty) and the assumed variability in the chosen probability distribution (aleatoric uncertainty). To obtain such a quantity, we look to Bayesian inference. Specifically, we can interpret GAMs fitted with penalised maximum likelihood in a Bayesian way and conduct Bayesian inference after fitting the model in mgcv.

### 2.3 Bayesian inference in GAMs

The fact that in GAMs we assume a-priori that all unknown functions of the covariates are smooth, can be viewed as a constraint on the values that β can take. This can be achieved this by assuming that β has the following prior distribution:

(7) $$\beta ∼N\left(0,S^{-}/\lambda \right)$$

where $S^{-}$ is the pseudo-inverse of the penalty matrix S (Wood, 2017, Wood, 2016). When the response is Normal (as in (4)) and we choose the prior $\beta ∼N\left(0,\sigma^{2}S^{-}/\lambda \right)$ (conditional on $\sigma^{2}$ for convenience), one can show (Wood, 2017) that the resulting posterior is

(8) $$\beta |y∼N\left(\overset{ˆ}{\beta },{\left(X^{\prime }X+\overset{ˆ}{\lambda }S\right)}^{-1}{\overset{ˆ}{\sigma }}^{2}\right)$$

where X is the model matrix induced by (3), $\overset{ˆ}{\beta }$ and ${\overset{ˆ}{\sigma }}^{2}$ are the maximum (penalised) likelihood estimates of β and $\sigma^{2}$, while $\overset{ˆ}{\lambda }$ is the estimate of λ. For distributions other than Normal, large sample approximations result in a posterior that is also multivariate Normal with a covariance matrix that depends on ϕ and λ (Wood, 2017). The R package mgcv readily provides these covariance matrices after the model is fitted.

With (8), we can now obtain the posterior predictive distribution (PPD) of any value of the response variable, say $\overset{\sim }{y}$, given the data via

(9) $$p\left(\overset{\sim }{y}|y\right)=\int_{\beta }p\left(\overset{\sim }{y}|\beta ,\lambda ,\phi \right)p\left(\beta |y\right)d\beta .$$

Note that although $p\left(\overset{\sim }{y}|y\right)$ integrates epistemic uncertainty and aleatoric uncertainty into one distribution, in our case there is a compromise. Not all epistemic uncertainty is “integrated” since we do not have posterior distributions for either λ or ϕ. As such, the integral in (9) is over β whereas for λ and ϕ we plug-in their likelihood estimates. We consider this compromise to be acceptable given the computational efficiency of using this approach.

In practice, we use Monte Carlo to approximate (9) by simulating multiple values from (8) and then for each such simulated value of β we simulate $\overset{\sim }{y}$ from its distribution (see Chapter 6 of Gelman et al. (2013)) — e.g. Normal with variance ${\overset{ˆ}{\sigma }}^{2}$ in (4). The 95% prediction intervals in Fig. 1d are computed as the 2.5% and 97.5% sample quantiles of such simulations.

### 2.4 Model checking

The ability to simulate from the PPD of y automatically enables probabilistic model checking (Gelman et al., 2013). The idea is simply to simulate many (e.g. N=1000) data sets using the same covariate values as in the original data y — i.e. simulate many realisations from p(y|y). Model checking then involves comparing the simulated data sets with the original one. For instance, we can compute a quantile (e.g. the median) of each of the N simulated data sets to obtain the predictive distribution of this quantile — and then check whether the sample quantile of the original data is extreme with respect to this distribution. If it is say, outside the 95% prediction interval, then we conclude that the model did not capture that particular aspect of the data well.

**Fig. 2:**
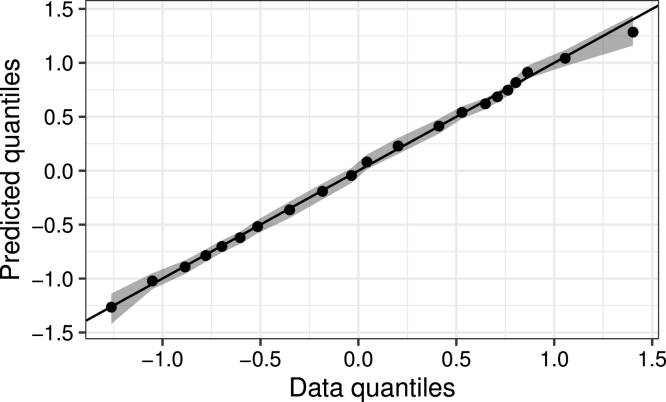
Quantile–quantile plot based on predictive simulations for the GAM in Fig. 1d.

Doing this for a sequence of quantiles values provides the equivalent of a Q–Q plot that compares the (marginal) probability distribution of the data with ones simulated from the model. Fig. 2 shows such a plot for the GAM in Fig. 1d, using 21 equidistant quantile values (0,0.05,0.1,…,1). The x-axis show the data quantiles while the y-axis show the mean of the PPD of each quantile, along with 95% prediction intervals. Intervals not containing the 45${}^{\circ }$ line indicate quantiles not well-captured. In this case all quantiles are captured well, indicating the model is a good fit. Note that such checking is not only probabilistic but it is also comprehensive as it checks the model as a whole, i.e. both the trend (5) and the assumed distribution (4). This way of model checking allows for user-specific properties of the data to be checked, including quantities not directly parameterised by the model such as autocorrelation structures and threshold exceedances (examples of which are shown in Section 3).

### 2.5 Moving window GAMs

**Fig. 3:**
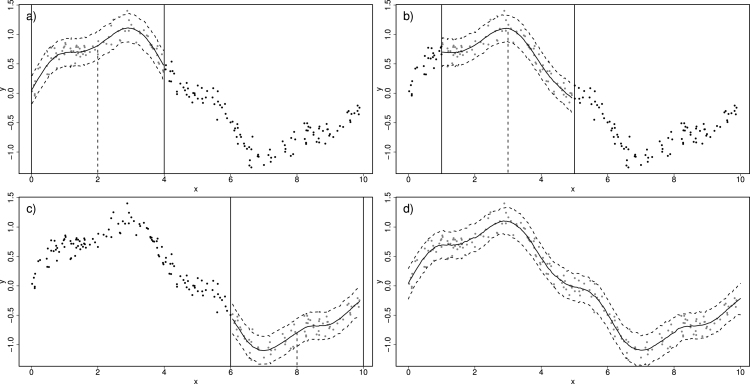
In (a), (b) and (c), an illustration of moving window Normal GAM. In (d) the moving window GAM is fitted on a grid of 200 equidistant x values.

We now go back to the idea of a moving window over space and time, within which a GAM is implemented and used to predict the value of the response. We start with the time dimension $x_{t}$. First, we investigate robustness to window size assuming some smooth “true” relationship has generated the data. Figs. 3a, b and c illustrate three instances where a Normal GAM ((4)–(5)) is fitted inside a moving window of size 4 units of $x_{t}$, on the same data from Fig. 1a. Notice how the prediction of the $y_{t}$ at the centre of each window is more similar to $y_{t}$ values closer to the centre compared to points further apart. To better deal with edge effects, we can use windows defined in terms of nearest-neighbours, and opting for 50 nearest-neighbours and rolling the window over 200 equidistant points in x, we obtain the prediction in Fig. 3d. The plot is essentially the same as the GAM fitted to the entire data set (Fig. 1d) which is to be expected when the “true” trend is smooth.

**Fig. 4:**
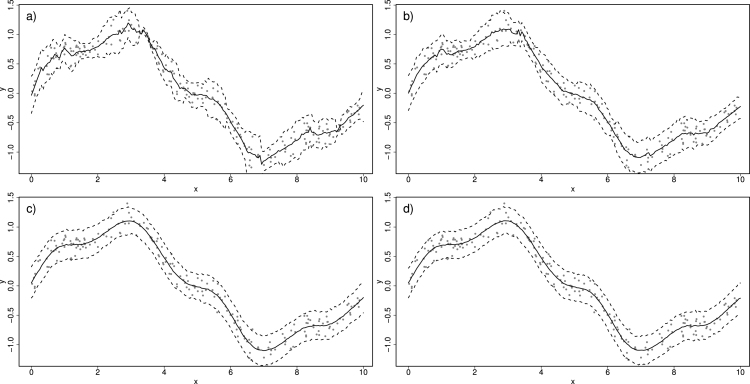
Illustration of the effect of window size, defined by 10, 20, 70 and 90 nearest neighbours as we move from (a)–(d) respectively.

To investigate robustness to window size, Fig. 4a–4d show the estimates from the moving window GAMs (MWGAMs) using a different number of neighbours, specifically 10, 20, 70 and 90. As expected, a too small window size (e.g. 10 and 20) results in predictions that are too wiggly. However as the number of neighbours increases (e.g. 70 and 90) the predictions stabilise (including the prediction interval) since the true trend is smooth. Naturally the threshold of how big window size needs to be is application specific, and therefore a requirement of using MWGAMs is some exploration of the window size prior to full implementation.

**Fig. 5:**
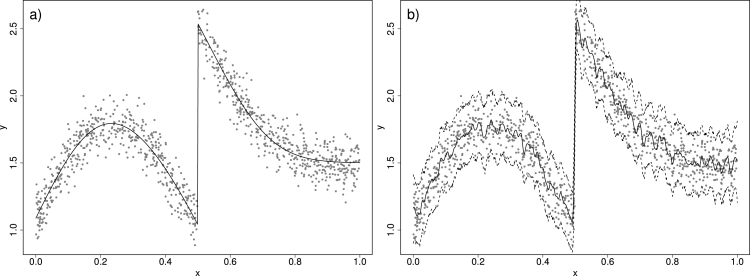
An example with discontinuity. In (a) the solid line is the true trend assumed to be the mean of a Normal distribution from which the data (in grey) are simulated. In (b) a Normal GAM is fitted to the data along with 95% prediction intervals.

We also consider the behaviour of the approach when discontinuities are present. A discontinuity, such as the one shown in Fig. 5a, will affect the smoothing properties of the predictions as the window size is increased. Large window sizes will allow discontinuities to affect the estimate of the penalty parameter λ when predicting data far from the discontinuity, and thus produce predictions that may not be as smooth as required. We illustrate by fitting a single GAM ((4)–(5)) to the data in Fig. 5a and present its predictions in Fig. 5b. Despite penalisation, the relationship is not as smooth as it should be since the discontinuity affects the amount of penalisation. In our moving window approach, because the underlying GAMs assume smooth relationships, discontinuities (or abrupt behaviour) such as the one depicted in Fig. 5a will tend to decrease the amount of penalisation (and thus smoothing) in the vicinity of the discontinuity.

**Fig. 6:**
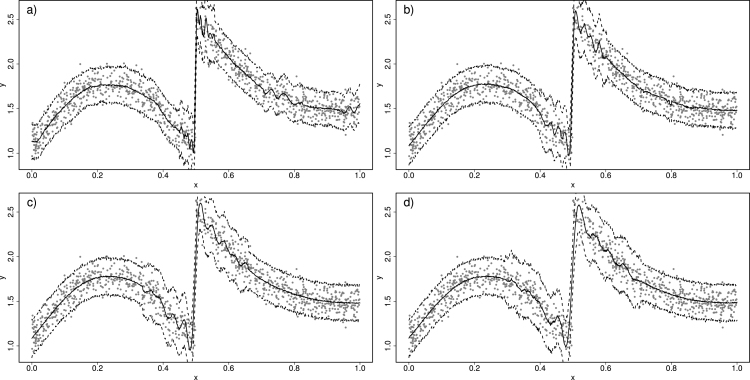
Illustration of the moving window GAM approach to the data from Fig. 5. Panels (a)–(d) illustrate the estimates when the number of neighbours ranges from 100–400 in steps of 100.

In other words, although we know that by increasing the window size we obtain a more optimal estimate of the underlying trend, we now have the potential problem that a too large window size might “under-smooth” the data if discontinuities are present. We suggest that a way to remedy this issue is to ensure that the auto-correlation structure in the data is adequately captured. Auto-correlation (or more generally dependency structure in $y_{t}$) will inevitably be underestimated if there is under-smoothing. We illustrate by example. In Figs. 6a–d respectively, we show predictions from the moving window approach when the number of neighbours increases from 100 to 400 in steps of 100. Notice how the estimated trend is less smooth near the discontinuity as the window increases. The prediction intervals are of course wider near the discontinuity as would be expected, but which window size do we choose?

**Fig. 7:**
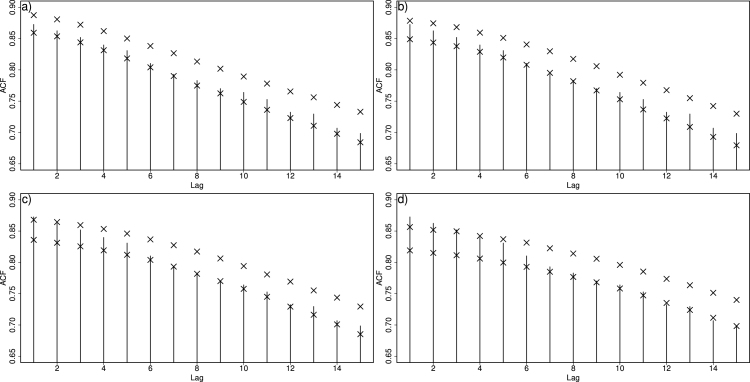
Autocorrelation function for each model in Fig. 6. The sample autocorrelation of the data is given in vertical bars while the 95% prediction intervals (symbols “×”) relate to the corresponding autocorrelation from data sets simulated by the model.

Fortunately, with simulations from (9), we can simulate 1000 realisations of the data and compare the autocorrelation of the simulations with the autocorrelation of the original data (as per Section 2.4). Specifically, we can produce 95% prediction intervals of the sample autocorrelation at different lags, and check whether they encompass the observed one. Fig. 7 shows the autocorrelation plots corresponding to Fig. 6. As window size increases, autocorrelation is underestimated by the data. Here, we would choose a size of 200 neighbours (top right panel) as the appropriate (yet subjective) compromise between flexible trend estimation and under-smoothing.

### 2.6 Higher dimensions

Extending the approach to covariates in more than one dimension is straightforward, since we can construct smooth functions of more than one variable using penalised splines (3). The most general approach is to construct tensor product interactions (Wood, 2017) where a smooth function of x and z say, can be constructed by making the coefficients of (3) smooth functions of z, e.g. 

(10) $$f\left(x_{i},z_{i}\right)=\overset{J}{\underset{j=1}{\sum }}\beta_{j}\left(z_{i}\right)b_{j}\left(x_{i}\right)$$

where $\beta_{j}\left(z_{i}\right)=\sum_{m=1}^{M}\gamma_{m}a_{m}\left(z_{i}\right)$ where $a_{m}\left(\cdot \right)$ can be of different basis than $b_{j}\left(\cdot \right)$. The same logic applies to higher dimensions, albeit at risk of heavy computational cost as the number of coefficients increases exponentially. A more efficient approach is to utilise thin plate splines, that can be constructed for any number of covariates — albeit under the assumption of isotropy. As such they are less flexible than tensor products since they will produce predictions that are invariant to covariate rotation (e.g., smoothness in space will only depend on distance and not direction). Both options are implemented in mgcv so choice depends on computational efficiency versus flexibility. Fortunately, with posterior predictive simulations this is a choice we can assess, as we demonstrate in the following spatial example.

**Fig. 8:**
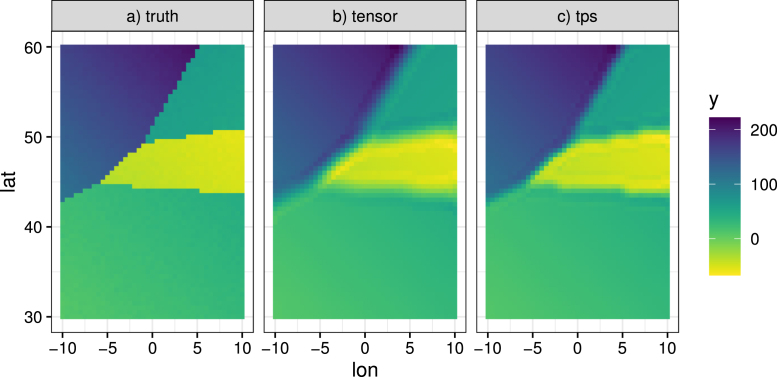
A spatial example. Data are simulated from a Normal distribution with a mean depicted by the spatial trend in (a).

**Fig. 9:**
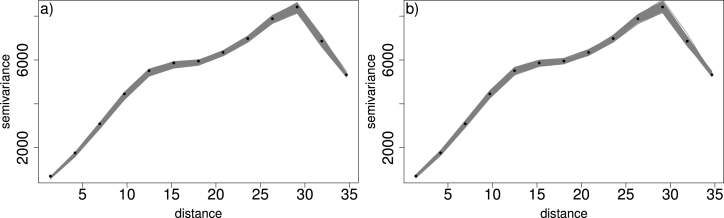
Empirical variogram (black points) of the data from Fig. 8a along with 1000 realisations of the variogram from simulations (which was clearer to illustrate than prediction intervals). Panel (a) relates to the tensor product model and (b) to the TPS model.

Fig. 8a shows simulated data where the true spatial trend is comprised of 4 contiguous regions where in each region we have a smoothly varying function of the coordinates (“lon” and “lat”), to emulate a situation with sharp discontinuities. This surface is assumed the mean of a Normal variable with constant variance, from which data are simulated. We apply the MWGAM (Normal distribution) to the data, assuming a window size of 50 nearest neighbours (Euclidean distance). In 8b the mean of each GAM was constructed by a tensor product of lon and lat with 16 knots in total, whereas in 8c we use a thin plate spline (TPS) also with 16 knots. The TPS is actually better at capturing the sharp discontinuities. We also check spatial autocorrelation by comparing the variogram (Chilés and Delfiner, 2012) of the simulated data with those from model predictions. Fig. 9 shows this for the tensor product and the TPS models. Both explain the spatial structure well, although the TPS captures the semivariance at lower distances slightly better. So in this case we choose the TPS model, given that it is also computationally faster.

### 2.7 Computational efficiency

The simplicity of the approach implies it is widely applicable to big data sets. It is by definition completely paralleliseable, and if the GAM in each window is given a small number of knots it is memory efficient and quick. In Fig. 6 for instance we assume 20 knots for each GAM costing 0.02 s per window on an Intel E5-1630 CPU at 3.70 GHz. For maximum modelling flexibility we can of choose as many knots as data points albeit at considerable computational cost. We recommend keeping the number of knots rather low (e.g. 20) and allow the window size to determine modelling flexibility.

In the spatial example of Fig. 8 we give the TPS model 16 knots so that each window costs 0.014 s resulting in computation time of 35 s for 2500 windows. Exploiting the machine’s 8 threads, the total run time is about 5 s with parallel processing. In addition, not requiring the entirety of the data for model fitting, means that implementing this approach is memory-efficient. In R for instance very large data sets can be dealt with by reading in portions of the data iteratively.

### 2.8 Dependence across variables

Suppose we have v=1,…,V variables $y_{v}$ to model. A further consideration when dealing with compound events is dependence across these variables, say $y_{1}$ (temperature) and $y_{2}$ (precipitation) beyond what can be explained by space and time. Up to a point, two (or more) variables will be correlated because of *when* they occur (e.g. summer) and *where* (e.g. near the coast). However some dependence may still exist (they are physically related after all). In our approach, we suggest using Gaussian anamorphosis (a technique popular in geostatistics, e.g. Chapter 6 of Chilés and Delfiner (2012)) to capture this dependence as follows.

The MWGAM approach can be used to capture the marginal distribution of each $y_{v}$ and produce the PPDs $p_{y_{v}}$ for each. We can use these to transform the original data onto a Gaussian (Normal) scale using probability integral transform (PIT). If $F_{y_{v}}$ is the distribution function of each $p_{y_{v}}$, we can transform each data point $y_{v,i}$ to obtain $u_{v,i}=F_{y_{v}}\left(y_{v,i}\right)$. Since in practice we obtain $p_{y_{v}}$ by simulation (Section 2.3), we do this using the empirical distribution function of the simulations. If the model is a good fit to the data, the $u_{v,i}$ will be Uniformly distributed on (0,1). We can then “put” $u_{v,i}$ on a Gaussian scale by

(11) $$z_{v,i}=\Phi^{-1}\left(u_{v,i}\right)$$

where Φ is the distribution function of the N(0,1) distribution, so that $z_{v,i}$ will be N(0,1).

In summary we can use our approach to transform the original data onto a continuous scale where the space–time structure captured by the MWGAMs has been factored out. Any dependence between the $y_{v}$’s not explained by space and time structures will be “buried” in the $z_{v}$’s. However, given that we can assume $z_{v}∼N\left(0,1\right)$, we can simply use correlation to quantify this dependence (using the V×V sample correlation matrix R). This is a special case of using Copulas (Joe, 2014) to describe dependence — here we effectively use the Gaussian Copula.

In practice, what this implies is that after we implement the MWGAMs, we compute the analogous correlation R of the transformed variables and then further simulate randomly from the multivariate Normal distribution:

(12) $$z^{\mathrm{sim}}={\left(z_{1}^{\mathrm{sim}},\ldots ,z_{V}^{\mathrm{sim}}\right)}^{\prime }∼MVN\left(0,R\right).$$

We then transform each simulated $z^{\mathrm{sim}}$ to the original scale via

(13) $$y_{v}^{\mathrm{sim}}=F_{y_{v}}^{-1}\left(\Phi \left(z_{v}^{\mathrm{sim}}\right)\right).$$

The predictions $y_{v}^{\mathrm{sim}}$ will have the space–time structure captured by the MWGAMs, but will also have the appropriate cross-correlation across variables.

## 3 Application to regional climate model data

We apply the approach to climate model output and illustrate the wide applicability of the MWGAMs by exploiting the plethora of available distributions in mgcv.

### 3.1 Data

The data are daily summer values (90 days between June and August) from 1545 land-based gridboxes over the UK mainland. The data come from a single ensemble member (number 1) of a regional climate model (RCM) of 12 km horizontal resolution used in the latest UK Climate Projections (UKCP) assessment (Lowe et al., 2018). The RCM UKCP projections were forced at the boundaries by an ensemble of the HadGEM3 global (60 km resolution) climate model (Williams et al., 2018). The projections use the RCP 8.5 scenario (Riahi et al., 2011) of future emissions, which dictate the levels of warming in the climate model. The data used are the model output re-gridded by the Met Office onto the Ordnance Survey Grid and are geographically labelled using Northings and Eastings.

The idea is to statistically model the projection data over two 11-year periods (1985–1995 and 2065–2075) and compare changes in compound events in the two periods, which are 80 years apart. We will model four variables: mean daily temperature, minimum daily temperature, daily precipitation total and daily mean relative humidity. For each 11-year period, there are 1,529,550 data points. To fully investigate changes to the climate, ideally 30-year length periods would be examined so that multi-decadal variability would be ‘averaged out’ to some extent, but here we are focusing on presenting the method using 11-year blocks of illustrative data.

The compound events that we are interested in are

1. Mean daily temperature exceeding 24 °C for 3 consecutive days (threshold used by the London Underground to take action for mitigating health effects from heat Public Health England (2018));
2. Seasonal average number of minimum daily temperature exceeding 10 °C and relative humidity exceeding 90% for two consecutive days (high chance of potato blight (Dancey et al., 2017, Garry et al., 2021));
3. Seasonal average number of warm–dry days, defined as days where mean temperature exceeds its historical period mean and precipitation is below its historical period mean (index of heat and drought risk (De Luca et al., 2020)).

### 3.2 Temperature models

For minimum and mean daily temperature we assume the Normal distribution so that in grid cell s and day t the model is

(14) $$y_{s,t}∼N\left(\mu_{s,t},\sigma^{2}\right)$$

(15) $$\mu_{s,t}=f\left(s,t\right)$$

where f(s,t) is a thin plate spline of easting, northing and day (all three scaled to be in [0,1]). Note the strong assumption of isotropy even across space and time — but recall that this GAM need only manage to adequately capture the space–time structure in small space–time windows and the fact that we can thoroughly check the model for violation of this assumption. The moving window grid size is 90 days ×11 years ×1545 grid cells ≈1.5 million.

**Fig. 10:**
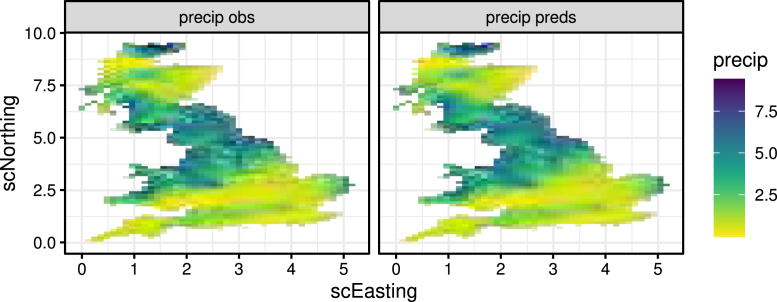
Left: map of precipitation from model output on 20th day of summer 1993. Right: posterior predictive mean of model predictions for the same day.

**Fig. 11:**
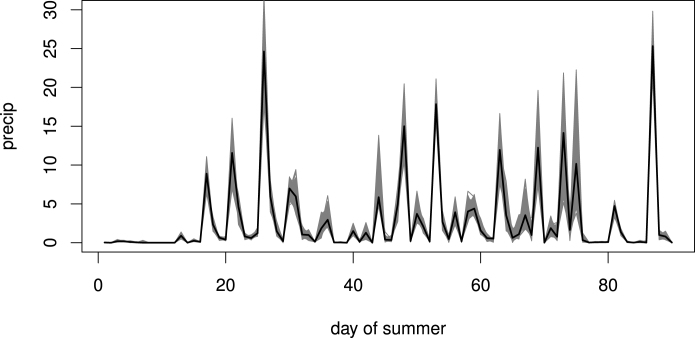
Black line: time series of daily precipitation in a randomly selected grid cell in 1993. Grey lines: 1000 corresponding realisations from the posterior predictive distribution.

### 3.3 Humidity model

Relative humidity (RH ∈[0,100]) is a percentage and so it is bounded. Here there are a few options, such as using the Beta distribution, however we opted for transforming RH using the logit function so we can assume the Normal distribution (noting that a Normal GAM is less computationally demanding than a Beta GAM). The model is

(16) $$\log\left(\frac{{\text{RH}}_{s,t}/100}{1-RH_{s,t}/100}\right)=y_{s,t}∼N\left(\mu_{s,t},\sigma^{2}\right)$$

with $\mu_{s,t}$ as in (15).

### 3.4 Precipitation model

Precipitation is more challenging since its marginal distribution has heavy tails and a mass of probability at zero, both properties being important as they relate to extremes. Given the challenge of finding appropriate transformations for zero-inflated variables, we opt for the Tweedie distribution, which arises when summing a Poisson distributed number of Gamma distributed variables. The Tweedie is parameterised by its mean μ and parameters θ>0 (scale) and π∈(1,2). It has a point mass at zero which becomes smaller as π→2 (with π=2 it reduces to the Gamma distribution). The model is

(17) $$y_{s,t}∼Tw\left(\mu_{s,t},\theta ,\pi \right)$$

(18) $$\log\left(\mu_{s,t}\right)=f\left(s,t\right).$$

### 3.5 Model implementation

We begin with the choice of a window size, which as before is defined in terms of number of neighbours. We start with an arbitrary number of spatial (e.g. 100 cells) and temporal (e.g. 15 days) “neighbours”, and also number of knots for f(s,t) (e.g. 50). Then we randomly choose a grid cell and a year (summer), and fit the model moving the window across the 90 days. Using posterior predictive simulations (Section 2.3) we check that (a) the Q–Q plot (Section 2.4) is adequate; and (b) the temporal and (c) spatial autocorrelation are well captured. If (a) is an issue, we can either change the assumed probability distribution or increase the number of knots. If either (b) or (c) are problematic, then we change the number of neighbours accordingly (noting that reducing the number of knots will generally increase the amount of autocorrelation). This is done for a number of randomly selected grid cells and years.

Following this procedure, we decided upon 5 days and 30 grid cells as a good choice of neighbourhood and 90 knots for f(s,t), for all four variables (min. and max. temperature, humidity and precipitation). It was interesting how the same window size seemed appropriate for all variables. Accounting for model fit and posterior predictive simulation, the most expensive model was the precipitation one which took about 6 h (for each 11-year period) using 8 threads in parallel on the same CPU described in Section 2.7.

### 3.6 Model adequacy

We start by visual inspection of model predictions compared with the data. For brevity we only show this for precipitation but note that similar checks were performed for the other variables. Fig. 10a shows the precipitation data for the 20${}^{\mathrm{th}}$ day of 1993 (both randomly selected), while Fig. 10b shows the mean of the corresponding PPD from the model. As expected, the predictions are smoother but capture the spatial structure well. Fig. 11 shows the time series of daily precipitation for a randomly selected grid cell in 1993, along with 1000 simulations from their respective predictive distribution. Note the increased variability for higher values, a general property of heavily skewed variables such as precipitation. Similar plots were produced for temperature and humidity to visually inspect that the space and time structures have been well captured.

**Fig. 12:**
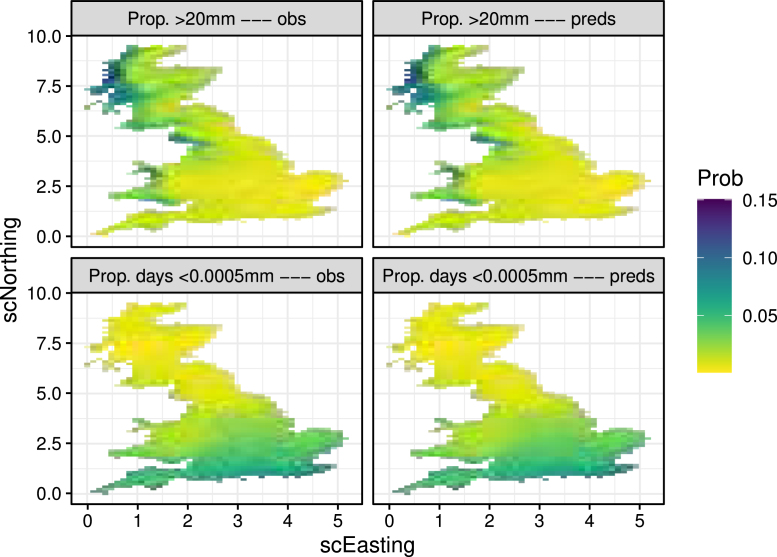
Proportion of days that precipitation is below 0.0005 mm (top) and above 20 mm (bottom) for the original data (left) and the predictions (right).

Moving window approaches will generally capture trends well so it is also important to check whether the distribution was appropriate. We can use the Q–Q plots suggested in 2.4 (shown in supplementary material Figure S1), however for 1,529,550 data points these can be quite misleading. Instead, we suggest specifying user-defined quantities designed to check particular aspects of the model. For instance, we can assess the two extreme ends of the precipitation distribution by checking how well the proportion of days less than 0.0005 mm and more than 20 mm (corresponding to the 2.5% and 97.5% sample quantiles of the data) is captured. Fig. 12 shows this for the historical period, indicating these extremes are well captured. (We also checked that the proportion of days with exactly zero rainfall is well captured, shown in Figure S2.)

**Fig. 13:**
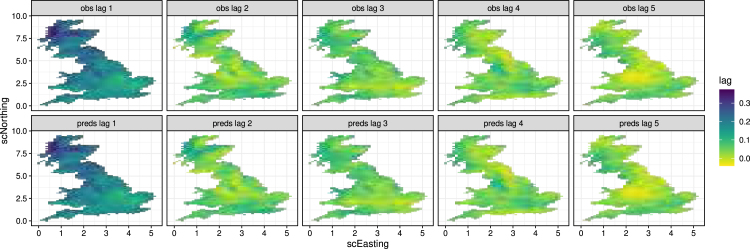
Sample autocorrelation of historical precipitation for lag up to 5 days. Top relates to original data, bottom relates to model predictions.

To check whether the amount of smoothing was appropriate, we check temporal and spatial autocorrelation. Fig. 13 shows maps of temporal autocorrelation for up to lag 5 indicating that this is well captured for historical precipitation. We also look at variograms in randomly selected grid cells to check spatial autocorrelation in the supplementary material (e.g. Figures S3 and S4).

**Fig. 14:**
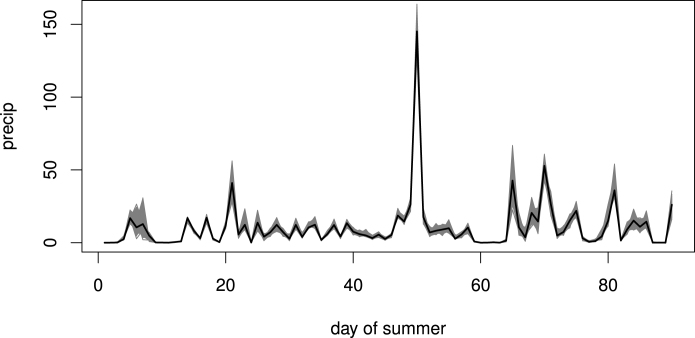
Out-of-sample predictions (simulations in grey) and corresponding precipitation data (black) for the summer that exhibited the highest precipitation value.

Lastly, we also perform out-of-sample predictive checks to more stringently test the model. For instance, we can leave out the grid cell with the highest precipitation value (across all grid cells and days) and check how well its values are predicted. Fig. 14 shows the precipitation time series for the particular summer within which this highest value occurred. The plot also shows 1000 realisations from the corresponding PPD, showing that this extreme has been well captured.

### 3.7 Cross correlation

Having modelled each of the four variables separately, we use the method in Section 2.8 to transform all of the data onto a N(0,1) scale and then compute the sample correlation in each grid cell. Note we need only do this for compound events involving more than one variable. Here looking at events 2., 3. in Section 3.1( , we only require the correlation between minimum temperature and humidity, and mean temperature and precipitation.

**Fig. 15:**
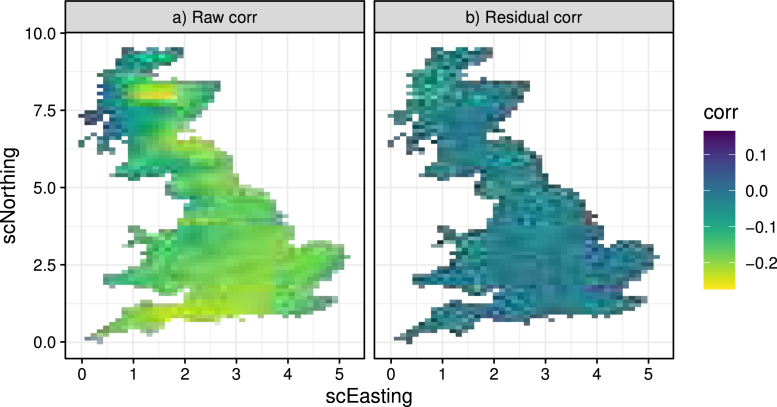
Left: sample (raw) correlation between mean daily temperature and total daily precipitation for the historical period. Right: residual correlation between the respective transformed variables.

Fig. 15a shows the raw correlation between mean temperature and precipitation whereas Fig. 15b shows the “residual” correlation between the transformed $z_{1}$ (temperature) and $z_{2}$ (precipitation). The plots indicate that almost all of the correlation is explained by the marginal models (i.e. space and time) since in Fig. 15b the values are small and exhibit no particular spatial structure. Nevertheless, we proceed by simulating $\left(z_{1}^{\mathrm{sim}},z_{2}^{\mathrm{sim}}\right)$ from a bivariate Normal distribution (12) to ensure that this residual correlation is propagated in the predictions.

### 3.8 Results

**Fig. 16:**
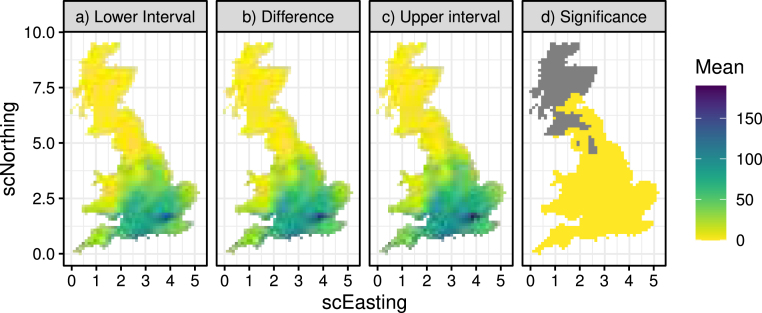
Panel (b) shows the difference between future and historical 3-day temperature exceedance above 24 °C, along with 95% prediction intervals (a and c) and an assessment of significance (d).

After ensuring that the models adequately capture the nature of the climate data, we can now investigate the future changes of the compound events of Section 3.1. Starting with event 1., Fig. 16b shows the difference between future and historical count of daily mean temperature exceeding 24 °C indicating that this clearly goes up in the future, particularly in the South-East. By checking whether zero lies inside the 95% prediction intervals of the difference (Fig. 16a and c) we can assess “significance”, shown in Fig. 16d. If zero is in the interval, then we conclude that the evidence is weak and depict this in grey.

**Fig. 17:**
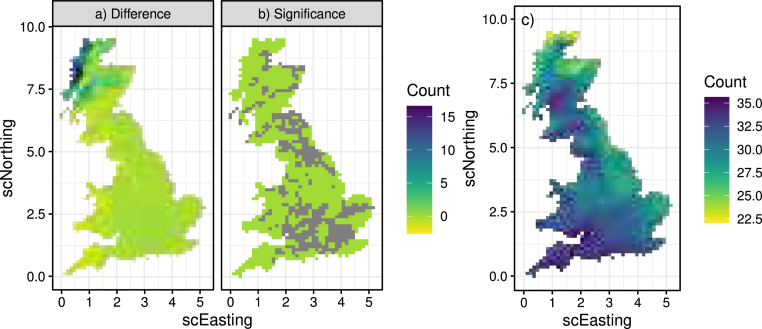
Panel (a) shows the difference between future and historical potato blight risk and an assessment of significance (b). Panel (c) shows the difference in number of warm–dry days.

Fig. 17a shows the difference in the seasonal average count of occurrences of event 2. along with a significance measure in Fig. 17b, indicating that this mostly goes up in the future, particularly in the North-West coast, although it also seems to go down in the Mid- and South-West coast. Finally, Fig. 17c shows the difference in the seasonal average count of occurrences of event 3. which is significant everywhere, and states that the number of such days in the summer will go up in the future — especially in the South- and Mid-West.

## 4 Conclusion

We proposed a practical approach based on statistical modelling, for quantifying future changes in compound events from climate model output. The approach is based on well-established statistical concepts and is thus transparent and interpretable. Predictions and estimates from this model can be thoroughly assessed, with straightforward uncertainty estimation of the predicted quantities. In addition, the fact that it is the raw weather variables that are being modelled, the approach is applicable to practically any compound event. Computationally the approach is very scaleable and parallelisable, so that the resolution of the model output is not a barrier.

We demonstrated the approach on data from a single ensemble member of a particular regional model, but for a variety of weather variables illustrating its flexibility and thus potential applicability to output from other physical models such as dispersion models. Having simulation-based predictions implies that the method can be applied in the same way to other ensemble members so that all simulations can be blended together. In this way, ensemble variability is also captured in the resulting predictions.

The same can be performed for multi-model climate data, where all simulations are put “in the same pot”. Given the Bayesian nature of the simulations however, one can additionally weigh the simulated predictions from each climate model on the basis of prior information regarding how “good” each climate model is. If the weights sum to one, then this is equivalent to Bayesian model averaging and in practice amounts to weighting the percentage of simulations to be included in the pot by the prior weights.

The approach does require certain user input, specifically in choosing a-priori the window size. We have presented a prescriptive way of choosing the window size, the uncertainty of which can always be propagated to the final simulations by repeating the modelling for various window-size choices and blending the simulations together. Nevertheless, future efforts will be focused on more elegant ways of choosing the window from the data — preferably during model fitting rather than a-priori.

## CRediT authorship contribution statement

**Theodoros Economou:** Conceptualization, Formal analysis, Methodology, Software, Writing – original draft. **Freya Garry:** Conceptualization, Data curation, Writing – review & editing.

## Declaration of Competing Interest

The authors declare the following financial interests/personal relationships which may be considered as potential competing interests: Theo Economou reports financial support and article publishing charges were provided by The Cyprus Institute.

## Data Availability

All the data, code and supplementary material are available and can be accessed at Zenodo (Economou, T., 2022) https://doi.org/10.5281/zenodo.6076085
